# Consensus on managing delayed methotrexate elimination in high-dose therapy: insights from the Middle East

**DOI:** 10.3389/fonc.2025.1660937

**Published:** 2025-11-06

**Authors:** Mubarak Al Manasour, Ahmad Absi, Ahmad Alhuraiji, Muhammad Faisal Khanani, Kayane Mheidly, Mohamed Elsaid, Walid Ballourah, Murtadha Al-Khabori, Naser Al Zain, Nisreen Khalifa, Rawad Rihani, Shaker Abdallah, Yasser Wali

**Affiliations:** 1 Adult Medical Oncology, Princess Noorah Oncology Center, Jeddah, Saudi Arabia; 2 College of Medicine, King Saud Bin Abdulaziz University for Health Sciences, Jeddah, Saudi Arabia; 3 Department of Hematology, Kuwait Cancer Control Center, Ministry of Health, Dasman Diabetes Institute, Dasman, Kuwait; 4 Department of Translational Research, Dasman Diabetes Institute, Dasman, Kuwait; 5 Pediatric Hematology Oncology, Tawam Hospital, Al Ain, United Arab Emirates; 6 Hematology Department, Sheikh Shakhbout Medical City, Abu Dhabi, United Arab Emirates; 7 Department of Pediatrics, King Faisal Specialist Hospital (KFSH) Madinah, Madinah, Saudi Arabia; 8 Department of Pediatric Hematology, King Faisal Specialized Hospital and Research Center, King Fahad Medical City, Riyadh, Saudi Arabia; 9 Department of Hematology, Sultan Qaboos University, Muscat, Oman; 10 Pediatric Hematology/Oncology, Mediclinic-Middle East, Dubai, United Arab Emirates; 11 Pediatric Hematology/Oncology, Mohamed Bin Rashid University of Medicine & Health Sciences, Dubai, United Arab Emirates; 12 Oncology & Stem Cell Transplant Dept., National Bank of Kuwait (NBK) Children’s Hospital, Ministry of Health, Kuwait City, Kuwait; 13 Department of Pediatrics, King Hussein Cancer Center, Amman, Jordan; 14 Department of Oncology, King Faisal Specialized Hospital, Jeddah, Saudi Arabia; 15 Child Health Department, College of Medicine and Health Sciences, Sultan Qaboos University, Muscat, Oman

**Keywords:** acute kidney injury, acute lymphoblastic leukemia, delayed methotrexate elimination, glucarpidase, hyperhydration, leucovorin, methotrexate, methotrexate toxicity

## Abstract

**Introduction:**

High-dose methotrexate (HDMTX) therapy is a cornerstone in treating pediatric and adult cancers, namely, acute lymphoblastic leukemia, non-Hodgkin lymphoma, and osteosarcoma, due to its capability to penetrate the blood–brain barrier. Despite its therapeutic benefits, HDMTX poses significant risks of delayed methotrexate elimination (DME) and associated toxicities such as acute kidney injury (AKI). These risks necessitate individualized dosing and preventive strategies, including hyperhydration, urine alkalinization, and leucovorin rescue.

**Methods:**

To address these challenges, a modified Delphi method with two rounds was used to develop consensus statements to guide clinicians in mitigating HDMTX-associated toxicities and optimizing management strategies. A panel of 13 experts from Saudi Arabia, United Arab Emirates (UAE), Kuwait, Oman, Jordan, and Egypt formulated 54 initial statements focusing on HDMTX regimens, risk factors, preventive care, and monitoring strategies.

**Results:**

Consensus (≥75%) was reached on 50 statements covering HDMTX regimens, preventive care, and toxicity management. Recommendations emphasized standardized methotrexate monitoring intervals, structured risk assessment for DME and AKI, supportive care measures (hyperhydration, urine alkalinization), pharmacokinetically adjusted leucovorin rescue, and the role of glucarpidase in severe toxicity or AKI.

**Conclusions:**

This consensus provides concrete clinical strategies for the safe and effective use of HDMTX, including structured risk stratification for DME, standardized monitoring intervals, pharmacokinetically guided leucovorin adjustments, and early glucarpidase intervention in patients with AKI or severe toxicity. These recommendations are particularly relevant for optimizing HDMTX administration in regions with limited access to advanced interventions.

## 1 Introduction

Methotrexate (MTX) is an effective, cost-efficient, and generally safe medication used to treat various hematological and oncological disorders, as well as autoimmune diseases (1). It is recognized as an essential medicine by the World Health Organization and is widely utilized globally for the treatment of various pediatric and adult cancers, including acute lymphoblastic leukemia (ALL), non-Hodgkin lymphoma (NHL), osteosarcoma, and medulloblastoma (2). High-dose MTX (HDMTX) is used in the treatment of a range of adult and childhood cancers (3). In this context, HDMTX is defined as the administration of MTX at doses of ≥500 mg/m² to achieve effective penetration of the blood–brain barrier (4).

While HDMTX is highly effective, a notable proportion of patients experience delayed MTX elimination (DME), which can significantly impact treatment safety and outcomes. HDMTX requires careful monitoring due to the potential for severe toxicities, notably acute kidney injury (AKI) from MTX crystallization in renal tubules, as MTX is primarily eliminated through the kidneys. This can cause DME, prolonging toxic exposure and increasing the risk of renal, hepatic, hematologic, and neurologic toxicities (5, 6). A recent study reported that among patients treated annually with HDMTX in France, Germany, Italy, and the UK, approximately 16% develop DME and around 9% may develop HDMTX-induced AKI (7). Thus, dosing must be individualized based on renal function, body surface area (BSA), and underlying comorbidities (8).

Preventive strategies, including hyperhydration, urine alkalinization, and leucovorin rescue, are vital to reduce the risk of DME and HDMTX toxicity (9, 10). Leucovorin provides an alternative source of tetrahydrofolate, which is downstream of dihydrofolate reductase, the enzyme inhibited by MTX (10). However, these interventions must be carefully balanced to preserve the pharmacokinetics and antitumor effects of MTX (3). Despite these measures, DME and eventual HDMTX toxicity cannot always be prevented and require prompt management.

Glucarpidase (carboxypeptidase G2) is recommended for patients with HDMTX exposure who exhibit dangerously elevated serum MTX concentrations and/or evidence of renal impairment, rather than for all individuals with general MTX-related toxicity. It converts MTX into inactive metabolites, glutamate and 2, 4-diamino-N10-methylpteroic acid (DAMPA), which are nontoxic and are eliminated through the urine or further metabolized by the liver (11). Early identification of DME is critical to facilitate timely interventions, such as intensified hydration, high doses of leucovorin, and glucarpidase administration, when necessary (3).

Patient responses to HDMTX vary significantly due to differences in pharmacokinetics; toxicity risk; and institutional practices in dosing, therapeutic drug monitoring (TDM), and supportive care. A recent systematic review highlighted the variability in dosing, sampling, and monitoring techniques, emphasizing the need for standardized TDM protocols to optimize MTX efficacy and safety (12). Moreover, individuals with Down syndrome (DS)-ALL, who are more prevalent in the Middle East, partly due to consanguineous marriages (13), have slower HDMTX clearance and are at higher risk of HDMTX-induced adverse events (AEs), even at lower doses than non-DS-ALL patients (14). Furthermore, the limited availability of TDM in many Middle Eastern centers further hinders individualized dosing and timely detection of DME, increasing the risk of AEs. These inconsistencies can lead to severe complications, including treatment delays and poor outcomes (15). Developing Middle East-specific guidelines is crucial to address regional variability, optimize treatment safety, and ensure consistent, evidence-based care.

This manuscript also addresses Middle Eastern-specific considerations, including pharmacoeconomic factors, variable availability of glucarpidase, and practical barriers such as infrastructure, laboratory access, and stockpiling policies, to enhance regional applicability. This modified Delphi consensus meeting aimed to address significant gaps in current practices, including the absence of evidence-based national guidelines and standardized approaches. Improvements in patient care are necessary to reduce morbidity and improve outcomes through the timely use of glucarpidase. This initiative’s primary objective was to develop a standardized protocol and regional consensus for the diagnosis and management of HDMTX toxicity, with a focus on the appropriate use and timing of glucarpidase administration to optimize patient outcomes.

## 2 Methodology

### 2.1 Methods for literature search

A targeted literature review was conducted to gather recent, high-level evidence on HDMTX treatment, toxicity management, pharmacokinetics, supportive care strategies (including hyperhydration, urine alkalinization, leucovorin rescue, and glucarpidase use), and patient-specific considerations. The literature search was undertaken in June 2024. Multiple electronic databases were systematically searched to gather relevant literature, including PubMed/MEDLINE, Embase, CINAHL, Web of Science, Scopus, and Google Scholar, to identify studies and guidelines relevant to developing consensus statements for HDMTX treatment in the English language. Medical Subject Headings (MeSH), free-text keywords, and Boolean operators ensured comprehensive retrieval of data.

The key words included “Consensus statement development, “ “Delphi method, “ “High-dose methotrexate (HDMTX), “ and related conditions such as “Primary central nervous system lymphoma (PCNSL), “ “Acute lymphoblastic leukemia (ALL), “ “non-Hodgkin lymphoma (NHL), “ and “Osteosarcoma chemotherapy.”

MeSH terms such as “Methotrexate, “ “Drug toxicity, “ and “Leucovorin” were used alongside expanded keywords such as “Methotrexate nephrotoxicity, “ “Glucarpidase in methotrexate toxicity, “ and “HPLC in methotrexate monitoring.” Search strategies included truncation, e.g., “methotrexate*, “ and Boolean combinations, e.g., “Consensus Statement*” OR “Delphi Method*” AND “Methotrexate” OR “High-Dose Methotrexate” AND “Toxicity” OR “Supportive Care.” Filters for human studies, clinical trials, reviews, and guidelines were applied where appropriate.

This was not a full systematic review, but a focused evidence synthesis intended to support statement development and ensure inclusion of the most relevant clinical trials, meta-analyses, and international guidelines.

### 2.2 Inclusion criteria

Studies focusing on participants from different populations with clinical diagnosis of HDMTX-related toxicities, including nephrotoxicity, neurotoxicity, mucositis, and hepatotoxicity, were included. Priority was given to studies that addressed toxicity management strategies such as supportive care measures, leucovorin rescue, urine alkalinization, hyperhydration, and the use of glucarpidase. Articles that evaluated HDMTX dosing, risk assessment, and pharmacokinetic monitoring were included, with emphasis on studies presenting measurable outcomes such as reductions in toxicity, treatment delays, or hospitalization duration. The eligible study types that were considered to ensure the inclusion of recent advancements encompassed peer-reviewed articles, systematic reviews, meta-analyses, randomized controlled trials, cohort studies, and expert consensus documents published in the English language within the last 10 years.

### 2.3 Exclusion criteria

Studies focusing exclusively on other chemotherapeutic agents, invasive interventions, or alternative dosing regimens without relevance to HDMTX toxicity were excluded. Non-peer-reviewed articles, opinion pieces without supporting evidence, gray literature, and studies with incomplete methodologies or inconclusive results were also omitted. Publications in languages other than English or those published more than 10 years ago were excluded unless they were foundational to the field. Duplicate studies, previously reviewed data, and articles with significant methodological biases or conflicts of interest were also excluded to maintain analytical integrity.

This targeted review did not aim to capture every published study but emphasized supporting expert judgment and facilitating informed consensus rather than conducting an exhaustive systematic review. Pre-reads were prepared and shared with the panel, followed by an online survey of consensus statements.

### 2.4 Panel generation

A diverse group of 13 specialists from adult and pediatric oncology, hematology, clinical pharmacology, and medical affairs participated in the consensus meeting on HDMTX toxicity (**Supplementary Table 1**). The panel was formed through an iterative and inclusive process to ensure a comprehensive and multidisciplinary approach. The members were selected to ensure broad expertise and geographic representation within the Middle East. Members represented Saudi Arabia, Kuwait, Lebanon, Syria, and the UAE, and were drawn from both academic and government institutions to minimize selection bias. All attempts were made to include representation from all relevant countries to ensure regional applicability. This diverse composition enhanced the panel’s credibility and the robustness of the consensus.

The panel was intentionally kept small to facilitate focused discussions, manageable iterative rounds, and high-quality expert input. Prior Delphi studies have shown that smaller panels can achieve valid consensus without compromising methodological rigor (3). All are experienced specialists in hematology/oncology or related fields, holding advanced qualifications and senior positions such as Professors, Consultants, and Section Heads. The panel included both adult and pediatric hematology/oncology, transplant, and internal medicine specialists, with most having over a decade of practice.

### 2.5 Steering committee

A steering committee was formed to guide the Delphi process, consisting of members who were experts in the subject matter and the Delphi methodology. Its role was strictly facilitative and non-voting, in line with best practices. It was convened to ensure methodological integrity and ensure transparency in guiding the Delphi process. The committee’s responsibilities included coordinating the literature review and drafting preliminary statements. Specifically, the committee defined the scope of the process, ensured all clinically relevant domains were represented, and oversaw the targeted literature review to support the evidence-informed statements. The members identified key areas for discussion and consensus, including the definition, severity, and diagnosis of HDMTX-related toxicities, development of guidelines and recommendations for managing these toxicities, and strategies for mitigating other treatment-related AEs. The committee drafted the initial survey instruments, coordinated survey distribution, collated quantitative and qualitative responses, and synthesized feedback for discussion without altering its substance.

### 2.6 Statement development

A total of 54 statements were formulated by the panelists and organized into six key sections: overview of HDMTX regimen, risk factors for DME and HDMTX toxicity, supportive and preventive care, monitoring strategies, emergent care for DME and HDMTX toxicity, and the use of glucarpidase. These sections collectively aimed to provide a comprehensive framework for the diagnosis, management, and treatment of HDMTX toxicity, drawing on the panel’s expertise to ensure that all critical aspects of the condition were addressed. Evidence levels were determined using the framework provided by the Oxford Centre for Evidence-Based Medicine. The quality of the supporting evidence was evaluated according to Oxford Centre for Evidence-Based Medicine: Levels of Evidence (March 2009) (16), based on the quality and type of supporting literature. For example, statements supported by systematic reviews or randomized controlled trials were assigned higher levels (e.g., 1a–1b), while those based on observational studies or expert opinion received lower levels (e.g., 3b–5). Pre-reads were prepared and shared with the panel prior to the online survey. The survey, comprising 54 consensus statements, was conducted to gather both quantitative and qualitative data from the panel.

### 2.7 Modified delphi methodology

The Delphi methodology was employed to gather expert consensus on the diagnosis and management of HDMTX toxicity. The modified Delphi methodology ensured a systematic, unbiased approach to building consensus by integrating expert opinions and evidence through iterative feedback. The process was conducted in two rounds to ensure comprehensive feedback and refinement of the consensus statements. A ≥75% agreement threshold was applied to establish consensus, in line with accepted Delphi methodology (17). The predefined thresholds for consensus were as follows: high consensus (≥75% agreement), moderate consensus (55%–74% agreement), and low consensus (<55% agreement). During consensus rounds, the steering committee facilitated structured discussions, clarified ambiguous wording, and ensured that all expert perspectives were fairly represented; however, it did not participate in voting or influence the content of the statements.

#### 2.7.1 Delphi round 1

In the first round of the Delphi online survey, statements were evaluated by the panel, and based on predefined thresholds, those that did not reach high consensus were categorized as having moderate to low agreement, thereby highlighting areas that required further clarification. The eight statements that initially achieved moderate or low consensus were primarily influenced by factors such as conflicting expert opinions, limited supporting evidence, or ambiguity in the original wording. The disagreements identified in Delphi Round 1 were addressed by rewording statements based on panelist feedback, followed by revision and re-presentation in the subsequent round.

#### 2.7.2 Delphi round 2

The revised statements, addressing areas of ambiguity and incorporating diverse perspectives, were presented in the second round of the modified Delphi process. This round included a physical meeting where the panel extensively discussed the statements, with moderate or low levels of agreement. The goal was to refine the statements to ensure that they reflected the collective expertise and practical considerations of the group. The final agreement, modification, or rejection of statements rested exclusively with the wider expert panel. The reframed statements were then revoted to finalize the consensus.

#### 2.7.3 Incorporation of panel feedback

In addition to quantitative voting, qualitative feedback was gathered through free-text responses and structured discussions. Key themes included requests for clearer definitions, concerns about redundancy, and differences in applicability across adult and pediatric populations. Statements were reworded for clarity, overlapping items consolidated, unsupported ones removed, and new statements added where gaps were identified (e.g., DS in pediatric patients). This iterative process ensured that revisions reflected collective input and enhanced the transparency and rigor of the consensus.

#### 2.7.4 Analysis plan

A 5-point Likert scale was utilized to gather participant responses (agree, strongly agree, neutral, disagree, and strongly disagree). Frequencies for each response category were recorded. No additional statistical analyses, such as measures of central tendency or dispersion, were performed, in line with standard Delphi consensus methodology. The panelists reviewed the qualitative data collected from the text box responses. After each round, the findings were discussed in a series of meetings to guide decisions on modifying, deleting, or adding statements.

**Figure 1:**
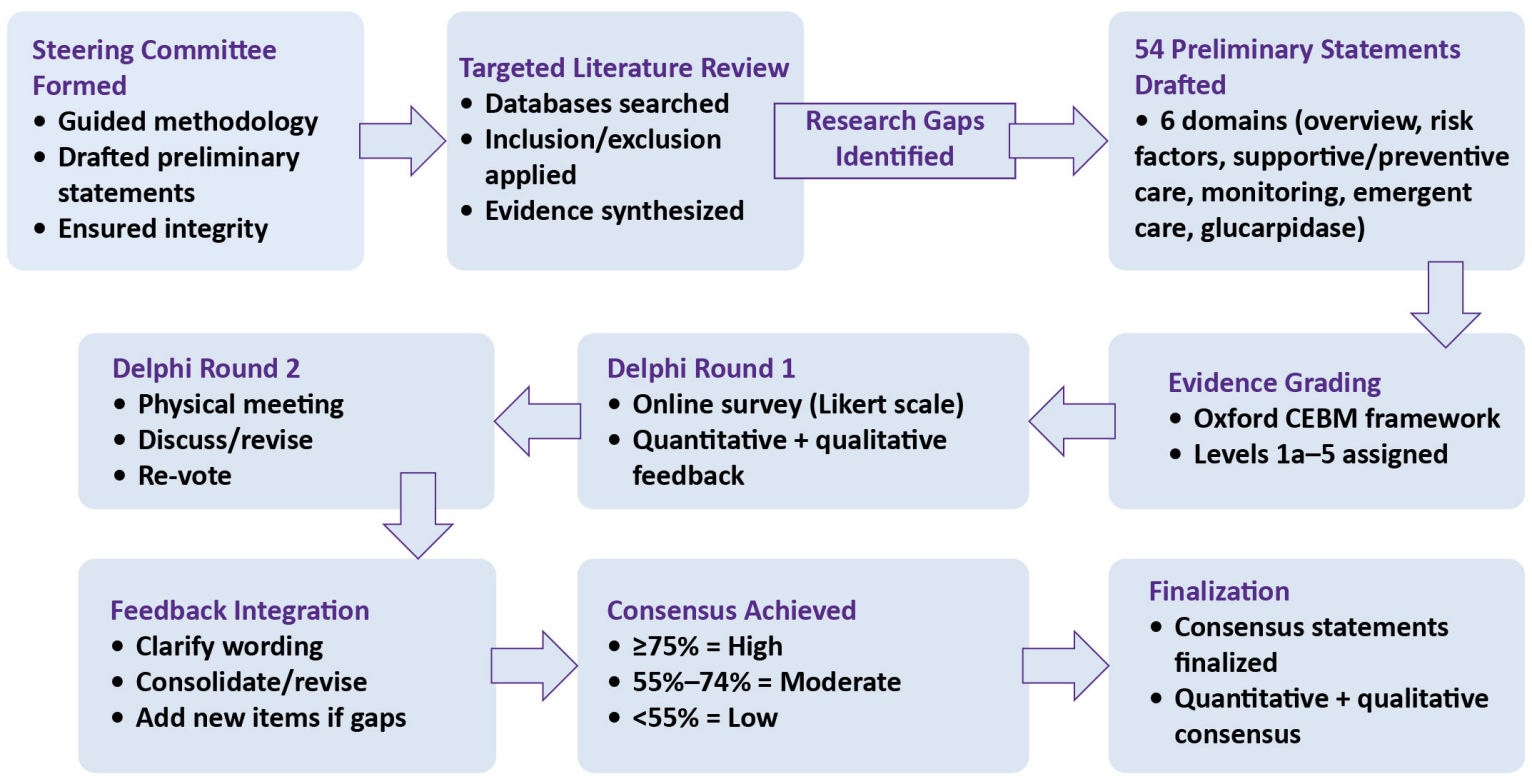
Pictorial representation of the employed modified Delphi consensus process.

An overview of the entire Delphi process is illustrated in **Figure 1**.

## 3 Results

### 3.1 First Delphi round

After the first Delphi round of anonymous survey, the overall agreement levels across the 54 statements were analyzed, revealing that after the first round, 46 statements achieved a high consensus (greater than 75%), while 8 statements reached moderate or low consensus. Specifically, statements 7, 10, 20, and 27 garnered low consensus, whereas statements 11, 15, 21, and 26 achieved moderate consensus.

### 3.2 Second Delphi round

To address these variations, several key modifications were made in the second Delphi round to refine the consensus. Statements 10, 11, 15, 21, and 27 were removed, while statements 7, 20, and 26 were revised to achieve a high consensus. Additionally, statements 13, 34, and 35, which had already attained high consensus in the first round, were slightly rephrased for enhanced clarity. A new statement addressing patients with DS within the pediatric population was also added. The initial statements, along with their corresponding levels of evidence and percentage agreement, are detailed in **Supplementary Table 2**. The total number of finalized statements after Round 2 was 50, as summarized in **Table 1**. The statements removed or revised in the second Delphi round were mainly refined for clarity and precision, without affecting key themes; the final 50 statements remain comprehensive and representative of expert consensus.

**Table 1:** Finalized consensus statements after the second Delphi round.

St no	Statements
HDMTX regimen overview	
O1	IV administration of HDMTX (≥500 mg/m²) is pivotal in treating PCNSL, ALL, and specific subtypes of NHL due to its ability to penetrate the BBB at these doses.
O2	MTX dosing and infusion times vary by diagnosis and patient characteristics, ranging from 0.5–5 g/m² over 4–36 hours in ALL to 8–12 g/m² over 4 hours in osteosarcoma.
O3	HDMTX remains the first-line treatment for ALL, NHL, and osteosarcoma and can be safely administered with supportive care measures (hyperhydration, urine alkalinization, high-dose leucovorin) to enhance MTX solubility and prevent toxicity.
O4	SCr level increase of ≥35.0 µM at 24 hours, 50% within 24 hours, or 25 µM/50% within 36 hours of HDMTX administration can be used as predictors for DME.
O5	HDMTX can be safely administered to patients with normal renal function through hyperhydration, urine alkalinization, and pharmacokinetically guided leucovorin rescue.
Risk factors for DME and HDMTX toxicity	
R1	Patient-level risk factors for DME include BMI ≥25 kg/m², concomitant medications, urine pH <7.0, IV fluid intake <3 L/m²/24 h, third-space fluid collections, hepatic dysfunction, renal insufficiency, and diarrhea. Having three or more of these factors is linked to significantly poorer survival.
R2	**Despite adherence to standard MTX protocols, patients receiving HDMTX may experience DME, prolonged TTC, and increased LOS.**
R3	HDMTX can cause severe toxicities, including nephrotoxicity, neurotoxicity, oral mucositis, neutropenia, and elevated liver enzymes; therefore, monitoring MTX levels and identifying risk factors are crucial.
R4	HDMTX administration results in AEs, with more than half of all patients experiencing mucositis and neutropenia.
R7	HDMTX toxicity may be increased by coadministration of drugs that displace MTX from serum proteins or reduce its clearance, particularly TMP-SMX and NSAIDs.
R8	**Presence of third-space fluids, such as pleural effusions or ascites, is a relative contraindication for HDMTX administration due to the risk of prolonged MTX half-life and toxicity; drainage of these fluids before treatment is recommended.**
R9	A high BMI is significantly associated with an increased risk of AKI in patients receiving HDMTX; therefore, BMI should be considered in risk assessment and management strategies for these patients.
R11	Patient age and BSA are significant predictors of MTX clearance, with their effects primarily influencing the distribution and elimination phases of MTX kinetics.
R12	Serum MTX and SCr levels are key parameters for identifying potential HDMTX-induced AKI.
R13	Pretreatment KPS and renal function may significantly impact the outcomes of HDMTX therapy.
R14	Any grade of renal dysfunction, including mild impairment (e.g., Cr clearance <60 mL/min), may increase the risk of toxicity during HDMTX treatment.
R15	**Furosemide has been identified as a risk factor for severe MTX-related renal toxicity and should be used cautiously when administering HDMTX.**
Supportive and preventive care	
S1	Supportive care must include measures to alkalinize urine and maintain adequate urinary flow to prevent MTX crystallization in the renal tubules.
S2	The hydration fluid should be supplemented with sodium bicarbonate to achieve a urine pH of ≥7; HDMTX should not be infused until this pH is reached.
S3	Hyperhydration (≥2.5 L/m² per 24 hours) with dextrose/saline and sodium bicarbonate should commence several hours prior to HDMTX administration and continue until MTX is cleared to nontoxic levels.
S4	Increased hydration may mitigate the impact of age and BSA on MTX clearance by enhancing renal elimination; therefore, hyperhydration prior to the first cycle of HDMTX for older patients and those with higher BSA may help prevent DME.
S5	**Loop diuretics or acetazolamide can be used to manage HDMTX toxicity to maintain diuresis and prevent fluid overload in patients with weight gain/fluid retention in select cases of severe renal impairment.**
Monitoring	
M1	UO within the first 24 hours has a significant impact on DME, TTC, and LOS in patients receiving HDMTX.
M2	An increase in SCr level within 24–36 hours after starting HDMTX may serve as an early indicator of DME.
M3	During HDMTX administration, maintaining UO at >100 mL/m²/h and urine pH ≥7 while avoiding weight gain is important.
M4	Cr and/or GFR should be monitored every 24 hours, starting 24 hours after HDMTX administration, with closer monitoring including cystatin C. if DME is suspected.
M5	Patient discharge can be considered when the serum MTX level is <0.1 µmol/L with stable renal function and electrolytes and no significant fluid overload or on Day 3 after HDMTX infusion if MTX kinetics at 48 hours are favorable (serum MTX level <1 µmol/L) and SCr levels are stable.
M6	Developing clinical decision support tools, such as MTXPK.org, can optimize model-informed prediction and timely intervention for DME before starting HDMTX administration by utilizing individualized patient data, including demographics, SCr levels, and real-time drug concentrations.
M7	**At 36 hours from MTX administration, leucovorin rescue should be initiated with 15 mg/m² for adults with low severity and 30 mg/m² for adults with moderate-to-high severity, and leucovorin should be discontinued when plasma MTX concentration drops below 0.1 µmol/L.**
M8	**Most laboratories use immunoassays to measure serum MTX levels; however, these methods do not reliably distinguish between MTX and its metabolites, and glucarpidase treatment may interfere with the results of these assays.**
M9	MTX levels are monitored using immunoassays, which are often conducted on automated analyzers; however, they incur high initial and recurring costs along with refrigeration requirements, making them complex and expensive.
M10	Chromatographic techniques are considered the gold standard for monitoring MTX levels, but they require significant initial investment, specially trained personnel, and have longer turnaround times for results.
Emergent care of DME and HDMX toxicity	
E1	Upon detection of HDMTX-induced AKI, initial supportive measures include urine alkalinization, fluid hydration, and high-dose leucovorin.
E2	Despite initial supportive care, if HDMTX toxicity persists, emergent care is necessary, which includes increased urine alkalinization, enhanced fluid hydration, and higher doses of leucovorin. Additionally, dialysis methods and glucarpidase may be considered.
E3	Patients with MTX toxicity receiving HFHD experience prolonged hospitalization, increased ICU use, high mortality, and significant MTX rebound after treatment, necessitating additional clearance sessions.
E4	Intensive HFHD can effectively clear MTX in patients with ESRD.
E5	Various leucovorin rescue protocols for DME are available, typically initiated 24–36 hours after MTX administration, with leucovorin administered every 6 hours at doses adjusted based on serum MTX levels.
Glucarpidase use	
G1	Glucarpidase is typically administered in cases of DME and HDMTX toxicity with renal deterioration, such as a >50% increase in SCr levels within 24–48 hours.
G2	Since glucarpidase mitigates the risk of acute renal failure by correcting DME in adult and pediatric cancer patients, its use allows for the continuation of HDMTX therapy without additional toxicity.
G3	Leucovorin, a substrate for glucarpidase, should be discontinued at least 2 hours before and resumed no sooner than 2 hours after glucarpidase infusion, continuing until serum MTX levels are undetectable.
G4	A dose of glucarpidase at 50 U/kg is effective and well-tolerated in pediatric, adolescent, and young adult patients with DME with or without renal dysfunction.
G5	MTX levels should be monitored after glucarpidase administration, ideally using an HPLC-based assay, until undetectable.
G6	In HDMTX-AKI patients, glucarpidase treatment within 60 hours significantly increases the odds of renal recovery, recovery from neutropenia, and normalization of liver enzymes, particularly enhancing renal recovery.
G7	Glucarpidase may be considered in cases of impaired renal function when plasma MTX concentrations are 2 standard deviations above the mean (as per MTXPK.org) or if plasma MTX levels exceed 50 μmol/L at 24 hours (only for HDMTx infusion duration less than 6 hours), 30 μmol/L at 36 hours, 10 μmol/L at 42 hours, or 5 μmol/L at 48 hours.
G8	Adult cancer patients treated with glucarpidase have lower inpatient and 90-day mortality rates than those who do not receive glucarpidase (including those on hemodialysis). They also have shorter overall hospital LOS and ICU stays.
G9	Administering glucarpidase within 72 hours of MTX administration significantly reduces the risk of severe MTX toxicity, with the recommended window being 48–60 hours after the start of HDMTX infusion.
G10	Timely glucarpidase use is more cost-effective than delayed administration or hemodialysis.
G11	Despite its effectiveness in lowering blood MTX levels, glucarpidase has minimal impact on intracellular MTX levels. Therefore, high-dose folinic acid must also be administered to manage intracellular MTX.
G12	Within 15–20 minutes of glucarpidase administration, MTX levels typically drop to 20% (as tested by immunoassay) and 1% (as tested by HPLC) of the original levels.
NS1	**In pediatric Down syndrome patients with ALL or lymphoma, HDMTX therapy is associated with increased rates of DME.**

Revised statements, based on the discussion, are shown in bold.

ALL: Acute lymphoblastic leukemia; AE: Adverse event; AKI: Acute kidney injury; BBB: Blood–brain barrier; BMI: Body mass index; BSA: Body surface area; Cr: Creatinine; DME: Delayed methotrexate elimination; ESRD: End-stage renal disease; GFR: Glomerular filtration rate; HFHD: High-flux hemodialysis; HDMTX: High-dose methotrexate; HPLC: High-performance liquid chromatography; ICU: Intensive care unit; IV: Intravenous; KPS: Karnofsky performance status; LOS: Length of stay; MTX: Methotrexate; NHL: Non-Hodgkin lymphoma; NSAID: Nonsteroidal anti-inflammatory drug; PCNSL: Primary central nervous system lymphoma; SCr: Serum creatinine; TTC: Time to clearance; TMP-SMX: Trimethoprim/sulfamethoxazole; UO: Urine output.

### 3.3 Domain-wise consensus summary

**Figure 2:**
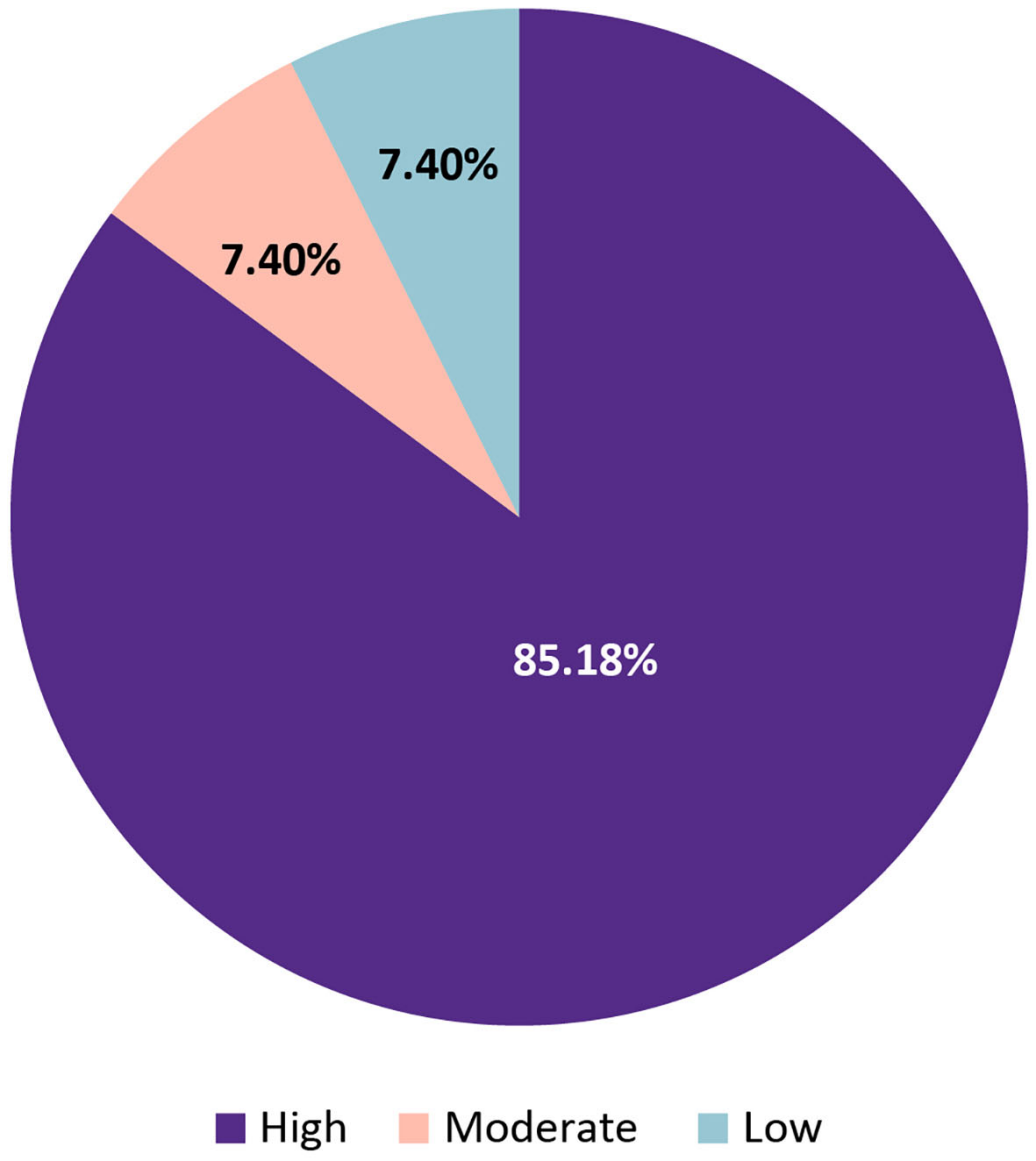
Overall distribution of consensus levels for 54 statements evaluated in Delphi Round 1 on the diagnosis and management of HDMTX toxicity.

Of the 54 statements evaluated in Round 1, 46 (85.2%) achieved high consensus (≥75% agreement), 4 (7.4%) reached moderate consensus (55%–74%), and 4 (7.4%) remained at low consensus (<55%) (**Figure 2**).

**Figure 3:**
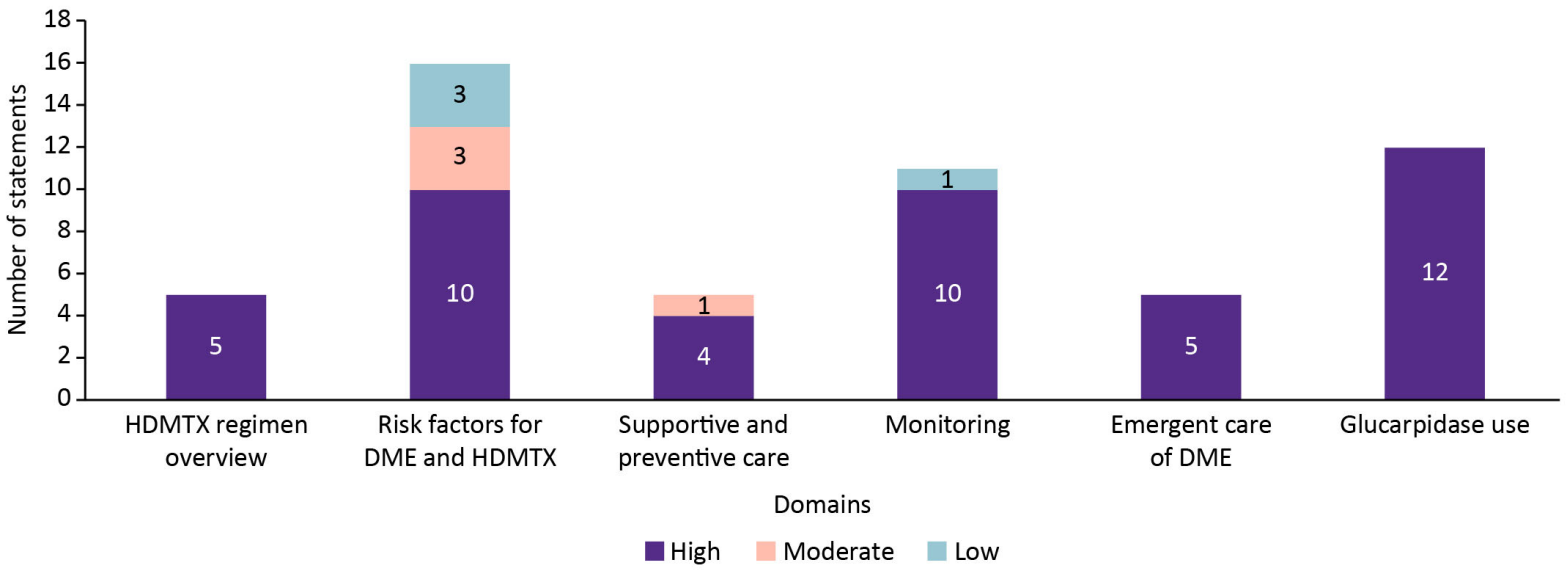
Consensus levels by domain for statements addressing HDMTX regimen, risk factors, supportive care, monitoring strategies, emergent management, and glucarpidase use.

By domain (**Figure 3**), all statements on the HDMTX regimen overview (5/5), emergent management (5/5), and glucarpidase (12/12) achieved high consensus. Most statements on risk factors (10/13), supportive and preventive care (4/5), and monitoring strategies (10/11) also reached high consensus, although moderate or low agreement was observed in the remaining statements within these domains. Overall, consensus was strongest for clinical management domains, while areas of lower agreement, principally risk factors and monitoring, were revised and re-presented for Round 2.

## 4 Discussion

### 4.1 HDMTX regimen overview

The expert panel reached consensus on several key aspects regarding the administration and management of HDMTX in the treatment of specific cancers.

Intravenous (IV) administration of HDMTX ≥500 mg/m² is crucial for treating primary central nervous system lymphoma (PCNSL), ALL, and certain NHL subtypes due to its capacity to penetrate the blood–brain barrier. The expert panel (92.3% agreement) emphasized that MTX, an antifolate, has long been a cornerstone of chemotherapy regimens for ALL and certain NHL subtypes (4). Its capacity to reach the central nervous system (CNS) also makes HDMTX effective in treating CNS lymphoma and leptomeningeal leukemia. Achieving therapeutic cerebrospinal fluid concentrations requires IV doses exceeding 1 g/m², given the approximate blood-to-cerebrospinal fluid MTX ratio of 30:1 (18) (Statement O1). This ratio is supported by pharmacokinetic studies rather than randomized clinical trials, which should be considered when interpreting its clinical relevance.

MTX dosing varies according to the specific therapeutic context. In non-oncological settings, weekly oral doses range from 2.5 to 30 mg, while doses of 20–40 mg/m²/week are combined with 6-mercaptopurine as maintenance therapy for ALL. In oncological settings, HDMTX (≥500 mg/m² IV) is employed for ALL, lymphomas, osteosarcoma, and as CNS prophylaxis in high-risk lymphoma cases. For low-risk lymphoma patients, HDMTX can replace cranial irradiation (3). This substitution is based on retrospective and expert-consensus evidence rather than head-to-head randomized comparisons.

The experts unanimously agreed (100% agreement) that MTX dosing and infusion schedules must be individualized based on the diagnosis and patient characteristics. For ALL, HDMTX is administered as either short (~3 hours) or prolonged (24–36 hours) infusions at doses of 1–5 g/m². In CNS lymphoma, short infusions (2–4 hours) of ≥3 g/m² per cycle are recommended (6, 19). For osteosarcoma, higher doses (8–12 g/m²) delivered over 4 hours are standard (19, 20). Further, in a recent study of 98 high-risk DLBCL patients receiving HD-MTX, 18.4% experienced delayed elimination, with none in those receiving 3-hour infusions; delayed elimination was associated with higher toxicity (77.8% vs. 26.2%, p < 0.05), suggesting shorter infusions may reduce complications (21). Additionally, short infusions of ≥3 g/m² MTX are employed as CNS prophylaxis in systemic lymphoma patients at increased risk of CNS relapse (22) (Statement O2). These recommendations primarily arise from retrospective clinical experience and expert consensus, with limited prospective trial validation.

HDMTX remains the first-line treatment for ALL, NHL, and osteosarcoma, with unanimous expert agreement. To maximize efficacy and minimize toxicity, it must be administered with supportive measures such as hydration, urinary alkalinization, urine pH monitoring, TDM and leucovorin rescue (15, 23). According to a multicenter survey, HDMTX was used to treat ALL in all study sites (100%), NHL in 16 sites (84.2%), diffuse large B-cell lymphoma in 9 sites (47.4%), osteosarcoma in 15 sites (78.9%), and medulloblastoma in 6 sites (31.6%). No other cancers were reported to be treated using HDMTX (Statement O3) (15, 23). It should be noted that while surveys provide valuable real-world data, they reflect practice patterns rather than controlled evidence of efficacy.

Renal function significantly impacts HDMTX safety. Even mild renal impairment (e.g., creatinine [Cr] clearance <60 mL/min) elevates the risk of toxicity. An increase in serum Cr (SCr) levels within 24–36 hours of initiating HDMTX can indicate DME. The experts (92.30% agreement) noted that a ≥35.0 µM increase in SCr level at 24 hours, a 50% increase within 24 hours, or a 25 µM/50% increase within 36 hours after HDMTX administration can predict nephropathy or dose modification needs (3) (Statement O4). These thresholds are based on observational evidence and consensus, and their predictive accuracy requires prospective validation.

HDMTX can be safely given to patients with normal renal function by employing hyperhydration, urine alkalinization, and pharmacokinetically adjusted leucovorin rescue, as unanimously agreed by the panel (100% agreement). Hyperhydration prevents MTX precipitation in renal tubules, reducing nephrotoxicity. Urinary alkalinization enhances MTX renal clearance by increasing the urine pH. These measures mitigate nephrotoxicity associated with pH-dependent MTX precipitation or tubular toxicity (24) (Statement O5). This approach is widely supported by expert consensus and clinical experience, although randomized data remain sparse.

### 4.2 Risk factors for DME and HDMTX toxicity

Monitoring serum MTX levels during hospitalization enables timely interventions to optimize clearance and reduce acute toxicity. Supportive care guidelines target specific timeframes for optimal MTX exposure and clearance, helping predict hospitalization durations and reducing healthcare costs (8).

#### 4.2.1 Risk factors for HDMTX toxicity

The majority of experts (92.30%) highlighted several patient-level risk factors that significantly impact the outcomes of HDMTX therapy. These included body mass index (BMI) ≥25 kg/m², concomitant medications (loop diuretics (e.g., furosemide), nephrotoxic agents (e.g., certain antibiotics), and anticonvulsants (e.g., levetiracetam), urine pH <7.0, IV fluid intake <3 L/m²/24 h, third-space fluid collection, hepatic dysfunction, renal insufficiency, and diarrhea. The presence of three or more of these factors is significantly associated with poor survival (25) (Statement R1). This association is based on retrospective evidence and requires validation in prospective cohorts.

Data on renal function (e.g., Cr clearance [CrCl]) cutoffs for HDMTX dose reduction or omission following prior injury are limited. However, some guidelines recommend dose reduction when CrCl is <50–60 mL/min and dose omission when it is between 10 and 30 mL/min (25). These thresholds reflect guideline-based consensus rather than robust trial-derived evidence.

A retrospective review of 447 HDMTX administrations in patients with leukemia and osteosarcoma identified key risk factors for delayed MTX clearance, prolonged time to clearance (TTC), and increased length of stay (LOS). These included urine output within 24 hours, renal toxicity, and the use of antiemetics such as metoclopramide, all of which contributed to longer TTC and LOS, highlighting potential areas for improved management (8). As this was observational, the findings identify associations rather than causality.

However, the experts noted that only a subset of patients receiving HDMTX under standard protocols experience DME, prolonged treatment, and increased LOS. The wording was refined to better reflect the frequency of these events while avoiding unnecessary patient quantification, accordingly (Statement R2) was modified after discussions (**Table 1**).

#### 4.2.2 Renal toxicity and associated risk factors

While HDMTX is generally well-tolerated, it can cause significant toxicity, including AKI in 2%–12% of patients. This nephrotoxicity arises from MTX crystallization in the renal tubular lumen, resulting in tubular damage (6). A prospective study conducted from April 2017 to October 2018 assessed HDMTX toxicity in 62 pediatric ALL patients. Among 244 HDMTX cycles, serum MTX levels exceeding 1.0 µmol/L in 35 cycles were associated with a higher incidence of toxicities, including oral mucositis, neutropenia, and liver enzyme elevation. Reduction and treatment delays were observed in patients with severe toxicities, highlighting the importance of monitoring MTX levels to manage risks (26) (Statement R3). This provides prospective evidence, although limited by a small sample size and a pediatric focus.

Furthermore, the incidence of renal toxicity associated with HDMTX in hematologic malignancies remains poorly characterized. In a retrospective study involving 649 cycles of HDMTX across 194 patients, renal toxicity was observed in 9.1% of cycles in patients with lymphoma, compared with 1.5% in those with sarcoma (27). This differential is notable, although the retrospective design limits interpretation.

Gastrointestinal (GI) toxicity is the main dose-limiting factor for MTX. Intestinal mucositis, a common side effect, causes nausea, abdominal pain, and cramping, often leading to malabsorption, weight loss, and treatment disruption (28). While most experts observed similar DME rates in lymphoma and osteosarcoma patients on HDMTX, a few noted higher rates in lymphoma patients. Despite differing opinions, most experts agreed that caution should be exercised regarding factors such as HDMTX dosage, patient weight, renal function, and tumor lysis rather than disease type. The group felt that the evidence was insufficient to definitively link disease type to DME, leading to the removal of Statement R5. This reflects consensus-based caution in the absence of strong comparative evidence.

**HDMTX-related toxicities**

HDMTX-related toxicities are classified as follows, aligned with the National Cancer Institute’s Common Terminology Criteria for Adverse Events (CTCAE) v5.0 (29).

- **Renal toxicity/AKI:** Defined as an increase in serum creatinine ≥1.5× baseline within 48 hours post-HDMTX infusion, often associated with delayed MTX elimination.
- **Hepatotoxicity:** Elevation of liver transaminases [alanine aminotransferase (ALT) or aspartate aminotransferase (AST)] ≥2× upper limit of normal, occurring within 7 days of HDMTX administration.
- **Hematologic toxicity:** Includes neutropenia (ANC <1.0 ×10^9^/L), thrombocytopenia (platelets <50 ×10^9^/L), and anemia (hemoglobin drop >2 g/dL), graded per CTCAE v5.0 criteria.
- **GI toxicity**: Grade ≥2 mucositis, nausea, or diarrhea occurring during or after HDMTX infusion.
- **Neurotoxicity:** Clinically significant neurologic events such as seizures, encephalopathy, or leukoencephalopathy temporally associated with HDMTX administration.

#### 4.2.3 Pediatric population and DS risk

A multicenter retrospective study on pediatric ALL patients (aged0–21 years) who received at least one dose of HDMTX from 2010 to2020 analyzed data on demographic and clinical variables extracted from electronic medical records. AEs such as mucositis, neurotoxicity, neutropenia, and thrombocytopenia were identified using algorithms based on Common Terminology Criteria for Adverse Events v5. The results showed that 86% of patients experienced at least one AE after receiving HDMTX, with half of the administrations resulting in an AE. More than half of the patients experienced mucositis and neutropenia. Older patients showed a higher incidence of certain AEs (30) (Statement R4). While informative, this study is retrospective, and causality cannot be assumed.

A retrospective study analyzed 269 HDMTX courses in 88 children with ALL or NHL. DME was defined as an MTX concentration higher than 1.0 mol/L at 48 h (31), occurred in 7.8% of courses. The risk factors included the first HDMTX course, low urine volume per BSA, high MTX dose, elevated bilirubin level, low estimated glomerular filtration rate, and reduced urine volume on the following day (32).

A retrospective analysis with 87 patients revealed that increased age and BSA should be considered risk factors for DME clearance following HDMTX infusions in pediatric patients. However, increasing the hydration rate to 200 mL/m²/h was shown to mitigate the impact of age and BSA on MTX clearance (24). Furthermore, in a recent retrospective study with 99 patients who received a total of 199 courses of HDMTX, DME was more frequently observed in patients aged ≥9 years, with a BSA ≥1 m², a BSA-based dose ≥4 g/m², and a MTX concentration ≥64 μmol/L at 24 hours (33). These results are hypothesis-generating but require confirmation in prospective studies.

However, the experts agreed that attributing a high DME risk to the pediatric population was misleading. Some suggested focusing on patients with DS who are known to have a higher DME incidence. They also recommended refining comparisons to improve clarity and suggested comparing DME incidence across different MTX regimens, leading to the removal of Statement R6. A previous study reported that children with DS and ALL experienced higher rates of severe toxicities, particularly with HDMTX. Among 103 DS and 1109 non-DS patients, DS patients had significantly more grade 3/4 toxicities with 5 g/m² HDMTX; however, reducing the dose to 0.5 g/m² lowered the toxicity without increasing relapse risk (34) (Statement NS1). This provides strong comparative evidence for differential toxicity in patients with DS, though derived from retrospective data.

#### 4.2.4 Impact of pharmacokinetic interactions

Pharmacokinetic interactions between drugs that are coadministered with MTX such as proton-pump inhibitors, β-lactam antibiotics, drugs that displace MTX from serum proteins or reduce its clearance such as trimethoprim/sulfamethoxazole (Bactrim^®^), and nonsteroidal anti-inflammatory drugs (e.g., indomethacin and naproxen) have been identified as causes of DME and subsequent toxicity (9, 35). Recent evidence indicates that prophylactic Bactrim^®^ does not significantly increase the risk of HDMTX-induced oral mucositis in children with ALL. However, clinical factors such as fever, skin rashes, neutropenia, AKI delayed MTX clearance, and higher 42-hour MTX levels have been identified as important contributors to mucositis risk (36). Other drugs, including pyrazoles, aminoglycosides, probenecid, certain penicillins, macrolides, and omeprazole, can also alter MTX elimination (35) (Statement R7). These interactions are supported by both mechanistic studies and case-based clinical observations rather than randomized evidence.

Additionally, the presence of third-space fluids, such as pleural effusions or ascites, prolongs MTX plasma half-life, increasing the risk of toxicity; therefore, draining these fluids before HDMTX is recommended (35). The experts agreed that while third-space fluids pose a risk of HDMTX toxicity, patients are typically treated cautiously with close dose adjustment/monitoring rather than being denied therapy. This resulted in a modification of Statement R8 (**Table 1**). The presence of third-space fluids should be ruled out before HDMTX, typically through sonography (9). This reflects consensus practice rather than prospective trial validation.

#### 4.2.5 Age and BMI in HDMTX toxicity

In a retrospective case–control study, a total of 302 patients received 840 infusions, with 8.6% requiring hospitalization. It was reported that BMI ≥23.8 kg/m² was independently associated with AKI after HDMTX (odds ratio: 3.8). Patients with a higher BMI had a greater risk of developing AKI, suggesting differential drug clearance in obese patients (4) (Statement R9). As this was a retrospective study, the strength of evidence is moderate and hypothesis-generating rather than definitive. Serum albumin acts as a carrier for MTX in the blood, and hypoalbuminemia is commonly observed in children with leukemia (37). In a prospective cohort study of 30 children with ALL, HDMTX was administered at doses of 2.5 g/m² for low-risk and 5 g/m² for standard/high-risk patients. The study found that preinfusion hypoalbuminemia (<3.5 g/dL) was significantly associated with increased grade 3–4 anemia, thrombocytopenia, febrile neutropenia, oral mucositis, and additional hospitalization due to HDMTX toxicity. Oral mucositis occurred more frequently in low-risk patients, while longer hospitalizations were observed in standard/high-risk patients. The findings suggest that optimizing serum albumin levels before HDMTX could help reduce toxicities (37). Although prospective, the small sample size limits generalizability.

The experts questioned the threshold for albumin levels in HDMTX toxicity. While hypoalbuminemia is a recognized risk, they agreed that the evidence was insufficient to establish a clear cutoff. A more general statement about hypoalbuminemia’s link to increased toxicity in adults was proposed, leading to the removal of Statement R10. This demonstrates expert caution in not overstating limited evidence.

In a recent retrospective chart review of 447 HDMTX administrations, univariate analysis revealed that both increased weight (relative risk: 1.003, 95% confidence interval [CI]: 1.0001–1.006, p=0.040) and age (relative risk: 1.03, 95% CI: 1.01–1.04, p<0.0001) were associated with prolonged TTC and LOS. Furthermore, multivariate analysis showed that age was the only demographic factor significantly linked to both increased TTC and LOS (8). This strengthens the role of age as an independent predictor but is still limited by a retrospective design.

MTX dosing is typically based on BSA estimates to account for body size-related variations in MTX clearance and volume of distribution. In a retrospective pharmacokinetic analysis of MTX plasma concentration data from hematological and oncological patients, it was concluded that factors such as age, sex, BSA, and SCr were significantly related to MTX clearance (Statement R11). Although BSA guides HDMTX dosing, renal function, age, hemoglobin, and genetic polymorphisms also significantly influence MTX clearance and toxicity risk. These pharmacokinetic findings are observational and need further validation in prospective pharmacology studies.

HDMTX therapy is feasible for most older patients (≥60 years) but should be initiated based on overall fitness and key risk factors, particularly renal function (3). HDMTX remains effective for PCNSL in patients aged over 80 years, provided that they have an adequate Karnofsky performance status (KPS) and renal function. However, reduced MTX doses are recommended for elderly patients with poor KPS or renal impairment (38). A recent study demonstrated favorable outcomes with HDMTX in elderly PCNSL patients who had a higher KPS, lower serum lactate dehydrogenase levels, and no deep brain involvement (39) (Statement R13). These findings are consistent across observational cohorts, although RCT evidence is lacking in elderly patients.

#### 4.2.6 Renal function and CrCl

Since approximately 90% of MTX is eliminated via the kidneys, AKI significantly impairs MTX clearance, exacerbating the severity of side effects. Therefore, preventing AKI in patients receiving MTX is crucial (40). Serum MTX and Cr levels are key indicators for detecting potential HDMTX-induced AKI (23) (Statement R12).

Lower CrCl prior to HDMTX administration is a predictor of renal toxicity, and both CrCl and SCr levels before infusion can help predict plasma MTX concentrations later (6). Although specific CrCl thresholds for dose reduction or omission of subsequent HDMTX have not been firmly established, dose reduction is typically recommended when CrCl is between 50 and 60 mL/min, and further HDMTX should be omitted if CrCl falls below 10–30 mL/min (3, 6) (Statement R14). These cutoffs represent consensus-based clinical practice rather than trial-validated thresholds.

In a study of 140 patients with 432 HDMTX exposures, the use of furosemide during HDMTX treatment was identified as an independent risk factor for nephrotoxicity (odds ratio: 2.56, p=0.001). This along with male gender, low albumin levels, and drug interactions increased the likelihood of developing nephrotoxicity, primarily of grade 1–2 severity (2, 41). While statistically significant, these findings come from observational data and require further confirmation. However, the experts agreed that furosemide, although a risk factor for HDMTX pharmacokinetics, should be used cautiously. It should be administered before rather than during HDMTX infusion to minimize the risk of DME. This led to the modification of Statement R15 (**Table 1**).

#### 4.2.7 Outpatient administration of HDMTX

HDMTX is traditionally administered in the inpatient setting. Supportive care, including vigorous IV hydration, urine alkalinization, and leucovorin use, significantly reduces morbidity. Monitoring serum MTX levels during hospitalization enables timely interventions to optimize clearance and minimize acute toxicity (8). According to a recent multicenter survey, administering HDMTX in the outpatient setting can improve bed utilization and reduce costs. As a survey-based study, these findings reflect practice patterns and feasibility rather than comparative clinical outcomes. While this approach was feasible, it was not widely adopted, with only two sites in the study routinely administering HDMTX as an outpatient procedure (2). The experts agreed that HDMTX is not typically administered in an outpatient setting due to the need for intensive monitoring and the high risk of serious toxicities, resulting in the removal of Statement R16. This position highlights a gap between emerging evidence supporting outpatient administration and current real-world implementation, where logistical and safety concerns remain paramount.

### 4.3 Supportive and preventive care

Vigorous hydration and urinary alkalinization are standard practices before and during MTX therapy. Since MTX is eliminated via the kidneys, maintaining high urinary flow rates and alkalinizing the urine enhance its elimination and prevent crystallization. These measures safeguard the kidneys and reduce the risk of other toxicities (42).

To minimize DME-induced toxicities with HDMTX, robust supportive measures are critical. MTX and its metabolites are poorly soluble at acidic pH; therefore, urine alkalinization and adequate urinary flow are essential to prevent crystallization. Hyperhydration with dextrose/saline (≥2.5 L/m²/24 h) should begin hours before HDMTX and continue until nontoxic levels are reached. The fluid should be supplemented with sodium bicarbonate (NaHCO_3_) to achieve urine pH ≥7 (3) (Statements S1 and S2). Moreover, a recent retrospective chart review showed that oral NaHCO_3_ when used with lactated Ringer’s solution is a viable alternative for urine alkalinization in MTX therapy. It requires lower total doses of NaHCO_3_ compared with IV administration, achieving comparable clearance times and pH levels without increasing AEs or delays in MTX elimination (43) (Statement S3).

Age and BSA likely influence the distribution phase of MTX kinetics, affecting elimination. Increased hydration improves renal elimination, reducing the impact of age and BSA on MTX levels at the 42- and 48-hour time points, with less effect at 24 hours. Hyperhydration before the first HDMTX cycle may prevent delayed clearance in patients with advanced age or high BSA (3, 24). In a retrospective study of 87 pediatric ALL patients treated with HDMTX (5 g/m² over 24 hours), increasing hydration from 125 to 200 mL/m²/h significantly reduced average serum MTX levels at 24, 42, and 48 hours, particularly in patients with delayed clearance. Hyperhydration improved renal elimination, lowering MTX and SCr levels, and nullified the predictive impact of age and BSA on delayed clearance (24) (Statement S4).

Loop diuretics should be administered to patients experiencing a quick onset of weight gain or edema to promote diuresis and prevent fluid overload. They could also be explored in order to support urinary flow in individuals with markedly reduced renal function (3). Acetazolamide, a weak diuretic that inhibits carbonic anhydrase in the renal proximal tubule, raises urine pH by increasing bicarbonate excretion. While not specifically approved for preventing HDMTX-induced nephrotoxicity, it may lower the risk of crystal nephropathy by reducing crystallization and enhancing MTX clearance through decreased tubular reabsorption. Interest in acetazolamide has grown during shortages of IV NaHCO_3_ (44). The experts agreed that loop diuretics are not mandatory for patients who experience weight gain or fluid retention during HDMTX therapy. However, they noted that loop diuretics may be considered based on individual patient needs and clinical management. In a recent study with 59 patients receiving 200 HDMTX courses, multivariate analysis identified loop diuretics or other diuretics with urinary acidification [Odds Ratio (OR): 4.91] as significant risk factors for AKI (45). Loop diuretics should therefore be avoided during HDMTX therapy due to their potential to increase AKI and MTX toxicity; if diuresis is necessary, renal function and urine pH should be carefully monitored. The decision to use diuretics should be guided by factors such as fluid status, kidney function, and the patient’s overall response to treatment, with the goal of optimizing care and minimizing complications. As a result, Statement S5 was modified (**Table 1**).

### 4.4 Monitoring

Regular monitoring of serum MTX and Cr levels after initiating HDMTX is crucial for detecting DME and enabling timely intervention to prevent DME-induced toxicity (3). MTX-related central neurotoxicity (MTX neurotoxicity) affects 3%–7% of children undergoing treatment for childhood ALL (46). The experts recommended removing statement M1, as it did not significantly influence the formulation of HDMTX toxicity and management guidelines.

A recent retrospective analysis identified several modifiable risk factors for DME, including urinary output in the first 24 hours, which significantly impacted delayed clearance, TTC, and LOS; renal toxicity, which was linked to prolonged TTC and LOS; and the use of antiemetic medications, particularly metoclopramide in the first 2 days, which correlated with increased TTC and LOS (8) (Statement M2).

Serum MTX levels should be monitored at standard intervals, such as at 24, 42, 48, and 72 hours, and repeated every 24 hours until the discharge criteria (MTX <0.1 µmol/L) are met. Renal function should be assessed at least once every 24 hours, beginning 24 hours after the HDMTX infusion, with more frequent checks if drug-induced nephropathy is suspected. Further evaluations should include clinical signs, fluid balance, body weight, urine output, and pH levels (3, 11) (Statements M3, M4, and M5), with elevated serum creatinine and MTX levels serving as secondary markers that should be closely followed. A recent study demonstrated that in both OS and ALL patients, the terminal phase of MTX elimination from 0.15 to 0.1 µM takes significantly longer than elimination from 0.2 to 0.15 µM. Also, when setting protocolized MTX threshold concentrations for discharge, it is important to consider the measurement method, as accuracy decreases at low MTX levels (47). Similarly, a recent study in children evaluated a single 72-hour MTX measurement at a regional cancer center. Higher doses (5 g/m²) increased diarrhea, thrombocytopenia, and hyperbilirubinemia, while 72-hour MTX levels did not predict toxicity. Delayed excretion was associated with elevated transaminases and creatinine levels. Single 72-hour monitoring with prolonged hydration and extended leucovorin rescue appears feasible, although its impact on treatment efficacy remains uncertain (48).

Discharge practices vary across centers. Some hospitals require patients to remain under strict clinical oversight until serum MTX levels are <0.1 µmol/L, renal function and electrolyte balance are maintained, with the patient in a stable clinical condition without significant fluid overload. In other centers, patients may be discharged by Day 3 after HDMTX infusion if MTX kinetics at 48 hours are favorable and Cr levels are stable (3) (Statement M6).

MTXPK.org is a web-based tool that can optimize model-informed prediction and timely intervention for DME before starting HDMTX administration by utilizing individualized patient data, including demographics, SCr level, and real-time drug concentrations (49). The experts emphasized that different ethnic groups exhibit varying toxicity levels at the same HDMTX dose, underlining the importance of considering ethnic differences in toxicity management (Statement M7).

HDMTX administration is routinely paired with leucovorin (folinic acid) rescue to mitigate toxicity. Leucovorin provides an alternative folate source to counteract MTX’s inhibition of dihydrofolate reductase. Rescue typically begins 24–36 hours after MTX infusion, with doses adjusted every 6 hours based on serum MTX levels (3). A retrospective study recommends initiating rescue at 36 hours, using 15 mg/m² for low-severity and 30 mg/m² for moderate- to high-severity cases, and discontinuing once plasma MTX levels fall below 0.1 µmol/L; doses are modified in delayed metabolism (50). Severity grading is based on MTX concentrations: low (1–10 µmol/L), moderate (10–100 µmol/L), and high (>100 µmol/L) (50). Experts emphasized calculating timing from HDMTX initiation and supported monitoring MTX levels at 24 hours for timely intervention, leading to the revision of statement M8.

Serum MTX levels are routinely monitored using immunoassays or chromatographic techniques. While immunoassays, commonly used in most laboratories, offer rapid results and high automation, they may be affected by cross-reactivity with MTX metabolites, such as glutamate and DAMPA (3). Chromatographic techniques, considered the gold standard, provide higher specificity and accuracy but require specialized equipment, trained personnel, and longer time-to-results (51). Detailed characteristics, advantages, and limitations of these assay methods are provided in **Supplementary Table 3**.

### 4.5 Emergent care of DME

As per a recent Delphi consensus, 90% of experts reported that nephrologists are typically involved in managing HDMTX-induced AKI. Other specialists, including intensivists, hospital pharmacists, neurologists, gastroenterologists, hepatologists, and endocrinologists, may be involved depending on the specific secondary toxicities (23).

Upon detecting HDMTX-induced AKI, supportive care measures are enhanced, including urine alkalinization and fluid hydration, along with the administration of high-dose leucovorin. If toxicity persists, treatment options primarily include dialysis-based methods and glucarpidase administration (23) (Statement E1). Some additional methods that can be employed include primarily hemodialysis, glucarpidase administration, and hemofiltration. Dialysis-based methods, though available, are slow and have limited efficacy. Prolonged exposure to toxic MTX serum levels is closely linked to the severity of systemic toxicities (23) (Statement E2). MTX is considered moderately dialyzable through intermittent hemodialysis. Long-term dialysis following MTX-induced AKI is rare. If extracorporeal treatment is necessary, intermittent high-efficiency hemodialysis is preferred. Hemodialysis, with or without hemoperfusion, is the most effective method for removing the highest percentage of MTX from the body over a given period (52). However, patients treated for MTX toxicity with dialysis or other supportive therapies often face prolonged hospitalizations and elevated mortality rates. In a study including Medicare beneficiaries diagnosed with PCNSL and treated for chemotherapy toxicity, patients requiring dialysis experienced even longer hospital stays, averaging 40 days (including 18 days in the intensive care unit), with a 90-day mortality rate of 59% (42) (Statement E3).

End-stage renal disease (ESRD) has traditionally been considered a contraindication to MTX therapy due to the heightened risk of serious adverse events (53). The high flux hemodialysis technique has been reported to effectively clear plasma MTX, enabling the administration of HDMTX to achieve complete remission with minimal and reversible direct MTX-related toxicity in ESRD patients (54) (Statement E4).

Rescue typically begins 24–36 hours after the MTX infusion starts, with leucovorin given every 6 hours at doses adjusted based on serum MTX levels. Leucovorin should not be administered earlier than 24 hours after the MTX infusion to prevent neutralizing MTX’s antitumor effects. Rescue continues until MTX levels reach nontoxic thresholds (3, 6) (Statement E5).

### 4.6 Glucarpidase use

Glucarpidase (carboxypeptidase G2 or Voraxaze^®^, BTG plc, London, UK) is a recombinant bacterial enzyme that deactivates MTX and folates by hydrolyzing their glutamate residues. It effectively reduces MTX levels by breaking it down into two nontoxic metabolites, DAMPA and glutamate, which are primarily excreted by the liver through bile rather than by the renal pathways (11, 55). Glucarpidase is typically administered when MTX serum levels are toxic and renal function deteriorates, such as when SCr levels increase by more than 50% within 24–48 hours (23) (Statement G1).

Glucarpidase rapidly reduces MTX serum levels by ~95% within 15 minutes (56). In a retrospective pediatric study (2012–2022), all 15 patients, mostly with ALL, achieved renal function normalization without grade ≥2 AEs, and subsequent HDMTX doses were well-tolerated (57) (Statement G2). While this study is small and retrospective, it provides clinically relevant evidence for efficacy and safety in children.

The approved dose is 50 U/kg IV bolus over 5 minutes, provided as 1000-unit lyophilized vials reconstituted with 1 mL 0.9% sodium chloride (3). Glucarpidase demonstrates consistent efficacy in complete MTX elimination (CIR) across ages 0–84 years, with no unexpected safety concerns (58) (Statement G4). A *post hoc* analysis of four compassionate-use trials in 86 patients showed median MTX reductions ≥98.7%, with higher CIR rates in those with pre-glucarpidase MTX <50 μmol/L (58). This study, although non-randomized, supports rapid and reliable MTX clearance, emphasizing the importance of early intervention.

Glucarpidase should be considered as early as possible, ideally within 48–60 hours after MTX infusion, based on plasma MTX levels, renal function, clinical symptoms, and risk of toxicity (3) (Statement G7). Recommended thresholds for administration are 50 µmol/L at 24 hours, 30 µmol/L at 36 hours, 10 µmol/L at 42 hours, or 5 µmol/L at 48 hours; it may also be used if MTX concentrations exceed the expected mean by ≥2 standard deviations, particularly in renal impairment. The pharmacokinetic tool MTXPK.org can guide individualized concentration–time assessment (49). Leucovorin should be continued per standard protocols until glucarpidase is given; if administered within 2 hours of leucovorin, both drugs are degraded, so leucovorin should be delayed at least 2 hours post-glucarpidase (3, 11) (Statement G3). MTX levels should be monitored continuously after glucarpidase, preferably using high-performance liquid chromatography (HPLC), until undetectable (Statement G5). HPLC monitoring up to 48–72 hours post-glucarpidase is recommended due to DAMPA interference with immunoassays for approximately 45 hours (11, 59) (Statement G12). As glucarpidase minimally affects intracellular MTX, high-dose folinic acid should be administered to mitigate intracellular toxicity (28) (Statement G11).

Although no head-to-head randomized controlled trials exist due to limited use, heterogeneous populations, and ethical constraints, observational studies support glucarpidase’s effectiveness (60). These studies are critically appraised for sample size, population, and relevance to timing, dosing, and monitoring recommendations. In the largest multicenter cohort of 684 adults with HDMTX-induced AKI (2000–2022), 207 patients (30.3%) received glucarpidase, while 477 (69.7%) did not. All treated patients received the drug within the recommended time window. Glucarpidase administration was associated with significantly improved outcomes, including higher rates of renal recovery, better neutropenia management, and normalization of liver enzymes, with a clear correlation between timely glucarpidase use and renal recovery (61) (Statement G6). While retrospective, this large cohort provides strong support for timely glucarpidase in adults with HDMTX-AKI.

A retrospective analysis of Medicare claims (2010–2017) showed that older cancer patients treated with glucarpidase had lower inpatient and 90-day mortality rates compared with untreated patients, including those undergoing hemodialysis. Additionally, glucarpidase use was associated with shorter overall hospital LOS and reduced intensive care unit stays (42, 60) (Statement G8). This claims-based study, despite potential confounding factors, reinforces clinical benefit in real-world practice and highlights cost-effectiveness considerations.

A combined analysis of four multicenter compassionate-use trials (1993–2007) evaluated IV glucarpidase in 476 patients with renal toxicity and delayed MTX elimination. Among 169 patients with baseline MTX >1 µmol/L, glucarpidase achieved a 99% median reduction in plasma MTX, with 59% showing rapid and sustained clinically important reductions. In patients with renal impairment (≥ grade 2), 64% recovered to grade 0–1 within 12.5 days. Glucarpidase provides effective, noninvasive rescue for MTX toxicity and may be beneficial even up to 90 hours after toxic MTX exposure, including in severe kidney injury. Exceeding the recommended therapeutic window should not preclude compassionate use when clinically indicated (62, 63) (Statement G9). The studies are critically appraised to confirm efficacy, even beyond standard time windows, informing compassionate use recommendations.

Optimal glucarpidase administration occurs within 48–60 hours of initiating HDMTX infusion, termed “timely glucarpidase” While administration after 60 hours (“delayed glucarpidase”) can still reduce plasma MTX, delayed use may not fully prevent life-threatening toxicities, as outcomes depend on both MTX concentration and timing (11). Timely administration is also more cost-effective than delayed glucarpidase or hemodialysis (60) (Statement G10).

Due to its infrequent use, glucarpidase is not typically stocked, with pharmacies requiring at least 24 hours’ notice (28). Regulatory frameworks, such as in the UAE, mandate stockpiling of critical agents, including leucovorin, glucarpidase, bicarbonates, and diuretics, to manage HDMTX toxicity efficiently (64). Additionally, the European Society for Pediatric Oncology recognizes HDMTX and glucarpidase as essential drugs for pediatric cancer treatment in Europe (65).

**Figure 4:**
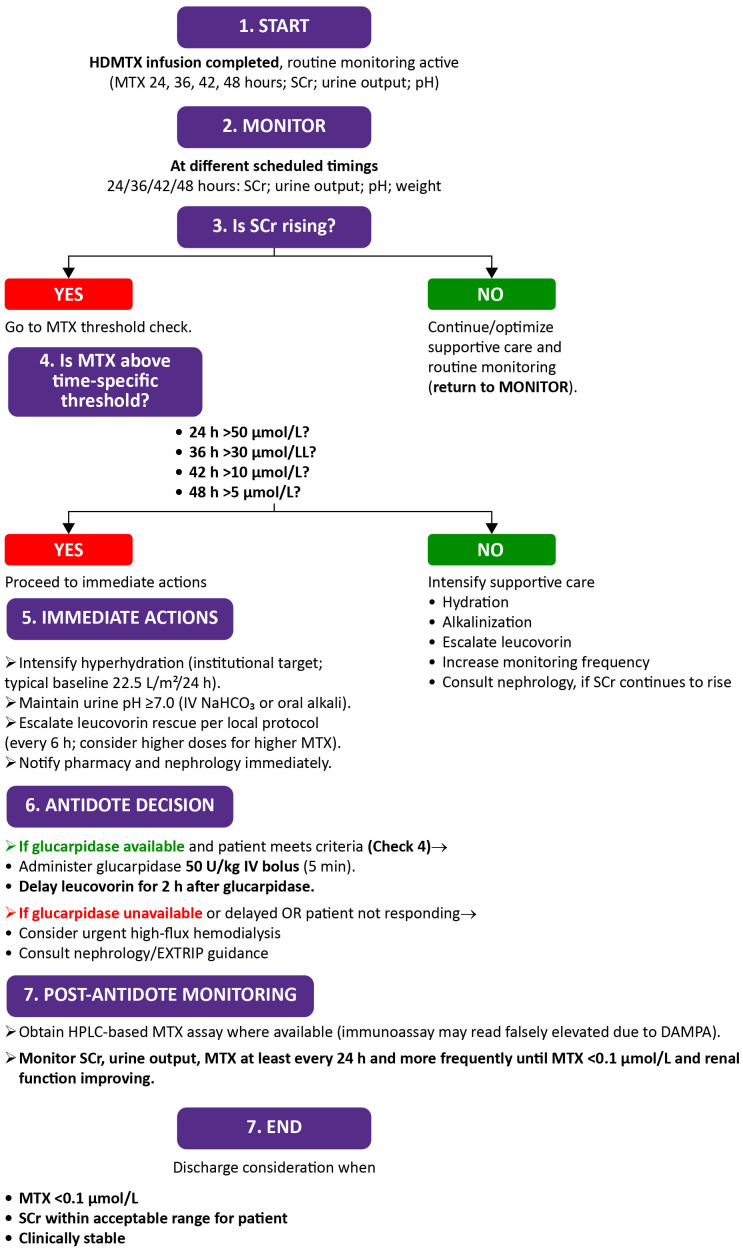
Algorithm for monitoring renal function and rescue strategies after high-dose methotrexate (HDMTX) administration. DAMPA, 4-deoxy-4-amino-N^10-methylpteroic acid; EXTRIP, Extracorporeal treatments in poisoning; HPLC, High-performance liquid chromatography; HDMTX, High-dose methotrexate; IV, Intravenous; MTX, Methotrexate; NaHCO_3_, Sodium bicarbonate; SCr, Serum creatinine.

The algorithm for monitoring and management after HDMTX, outlining serial monitoring of renal function and MTX levels, with supportive interventions such as hydration, alkalinization, leucovorin escalation, and glucarpidase when indicated, is illustrated in **Figure 4**.

**Table 2** provides a comparative overview of HDMTX management strategies across different guidelines and the present Middle East Consensus. This comparison aims to support clinicians in understanding regional adaptations and globally accepted best practices for HDMTX administration and toxicity mitigation (66, 67).

**Table 2:** HDMTX management as per NCCN and SIOPE guidelines and the present consensus.

Aspect	NCCN guidelines (66)	SIOPE guidelines (67)	Middle East consensus
Patient selection	Emphasizes pretreatment evaluation of renal function, hydration status, and avoidance of third-space fluids.	Requires pretreatment evaluation of renal function and urine output. Includes recommendations for high-risk patients (e.g., with low BSA, Down syndrome, etc.).	Expanded checklist-based risk stratification, including: BMI ≥25 kg/m², hypoalbuminemia, creatinine clearance <60 mL/min, diarrhea, and drug interactions. Patients with ≥3 risk factors considered high-risk; recommends pre-infusion optimization (e.g., albumin correction, fluid drainage).
Hydration protocol	≥2.5–3.0 L/m²/day with NaHCO_3_; initiate 12 h before MTX.	At least 3 L/m²/day IV hydration; initiate 6–12 h prior to MTX; titrate to achieve urine pH ≥7.	≥2.5 L/m²/24 h hyperhydration with dextrose/saline + NaHCO_3_; allows oral NaHCO_3_ in shortages/resource-limited settings; recommends escalation triggers if urine output <100 mL/m²/h or urine pH <7; suggests pre-cycle intensified hydration in elderly, obese, and high-BSA patients.
Urine alkalinization	NaHCO_3_ to achieve urine pH ≥7 before starting MTX.	Mandatory alkalinization with NaHCO_3_. Urine pH ≥7.0 before infusion.	Same urine pH ≥7 target, but permits oral or IV NaHCO_3_; includes algorithm for checking urine pH every 2–4 h; recommends acetazolamide if IV NaHCO_3_ unavailable; emphasizes correction of metabolic acidosis prior to infusion.
Leucovorin rescue	Starts 24 h after infusion; dose guided by MTX levels.	Starts 24–36 h after infusion; individualized rescue dose based on severity and MTX levels.	15–30 mg/m² q6h; introduces dose-banding by severity: low (MTX 1–10 µM), moderate (10–100 µM), high (>100 µM). Adjust per MTX levels; resume ≥2 h after glucarpidase; provides explicit severity categories for decision-making.
Monitoring of MTX	Levels at 24, 48, 72 h; discontinue when <0.1 µmol/L.	Standards at 24, 48, 72 h; recommends model-based tools (e.g., MTXPK.org).	Routine 24-h sampling minimum; increase frequency if MTX rising or renal deterioration; monitor creatinine, electrolytes, fluid balance, urine output, body weight daily; recommends MTXPK.org; HPLC preferred after glucarpidase due to DAMPA interference; discharge criteria defined: MTX <0.1 µM, stable renal/electrolytes, no overload.
Glucarpidase use	For DME with renal dysfunction or MTX levels above protocol thresholds.	Same as NCCN; indicates use when plasma MTX >2 SD above population mean.	50 U/kg single IV dose; consensus threshold: MTX >50 µM at 24 h or renal deterioration (differs from NCCN cutoffs); administer within 48–60 h, but compassionate use allowed even later; leucovorin withheld 2 h post-dose; HPLC monitoring up to 72 h due to DAMPA interference.
Resource adaptation	Assuming availability of high-resource tools.	European-center dependent; strong reliance on institutional infrastructure.	Adaptations include oral NaHCO_3_, simplified monitoring if assays delayed, loop diuretics for fluid overload, escalation criteria for referral; UAE mandates stockpiling of leucovorin, glucarpidase, bicarbonates, and diuretics.
Novel consensus-specific contributions	–	–	Checklist-based risk stratification, severity-based leucovorin dosing bands, algorithmic urine alkalinization (oral NaHCO_3_/acetazolamide), escalation triggers, and UAE policy stockpiling.

BMI, Body mass index; BSA, Body surface area; DME, Delayed methotrexate elimination; HDMTX, High dose methotrexate; HPLC, High-performance liquid chromatography; IV, Intravenous; MTX, Methotrexate; MTXPK.org, Methotrexate Pharmacokinetics Online Tool; NaHCO_3_, Sodium bicarbonate; NCCN, National Comprehensive Cancer Network; q6h, Every 6 hours; SD, Standard deviation; SIOPE, European Society for Paediatric Oncology; UAE, United Arab Emirates.

This consensus paper benefits from a multidisciplinary approach, evidence-backed statements, and a strong focus on clinical applicability. However, future research should address the lack of large-scale, prospective studies to validate these recommendations. Exploring pharmacogenomic profiling could optimize HDMTX therapy, enabling more personalized treatment, while investigating new therapeutic adjuncts may help reduce toxicity and improve patient outcomes in HDMTX management. A recent study found that the AA genotype of MTHFD1 rs2236225 was associated with grade III–IV GI toxicity (p=0.03), while the A allele of MTHFR rs1801133 (p<0.01) and AA genotype of GSTP1 rs1695 (p=0.02) were linked to grade I–IV hematologic toxicity (68). These findings support the use of genetic biomarkers to guide safer, individualized HDMTX dosing. Further, in 713 Chinese PCNSL patients receiving 3021 HDMTX courses, higher albumin levels and certain genetic variants (ABCB1 rs1045642, MTHFR rs1801131, MTHFD1 rs2236225) were linked to lower risk of hematologic and hepatotoxicity, while female sex and co-use of loop diuretics or levetiracetam generally increased toxicity risk. Specific SNPs (rs1801133 GG, rs1128503 GG/AG, rs2231142 AA/AC, rs717620 TT/GT) elevated toxicity risk, whereas rs1045642 TT and rs1801394 GG/AG were protective. Overall, albumin levels and medication/genetic profiles are key predictors of HDMTX-related toxicity (69). Additionally, the genetic variation between individuals further suggests that DME and HDMTX toxicity remains unpredictable, even with extensive standard of care measures. Beyond pharmacogenomics, future research should explore novel therapeutic adjuncts, establish plans for periodic updates of these consensus statements, and evaluate strategies for integrating them into national policies and regional practice frameworks to ensure their long-term applicability and impact.

Individual statements evolved throughout the process, reflecting ongoing discussions and the integration of new evidence on various aspects of HDMTX toxicity, risk factors, and management strategies. Nevertheless, this consensus is subject to potential limitations in the context of HDMTX management. The consensus is context-specific, shaped by particular clinical settings, and may not be generalized across different hospitals or patient cohorts. Furthermore, potential biases from conflicts of interest or preexisting beliefs could have influenced the recommendations; however, independence was safeguarded through a structured literature review, transparent voting, and collective expert judgment. Lastly, variability in clinical practice and healthcare resources can affect the consistency and implementation of these guidelines across diverse settings. Immediate actionable steps to address the limitations of the consensus include implementing structured training programs to reduce inter-clinician variability, developing standardized local protocols based on the consensus recommendations, and encouraging pharmacovigilance reporting to monitor real-world variations and safety concerns. Collectively, these measures provide practical improvements in HDMTX management, while longer-term studies and pharmacogenomic investigations are being pursued. Despite these limitations, this consensus provides a strong foundation for further exploration of HDMTX management strategies, emphasizing the need for more comprehensive studies and broader inclusion of diverse perspectives in future research and guideline development.

## 5 Conclusions

This consensus on HDMTX toxicity management emphasizes evidence-based, patient-centric approaches, with a strong focus on clinical applicability and multidisciplinary collaboration. It highlights the critical role of standardized protocols in improving outcomes, especially in high-risk settings, while addressing the diversity of practices between institutions. The goal was to develop a standardized, region-specific protocol for diagnosing and managing HDMTX toxicity, with a strong emphasis on glucarpidase use.

Over the consensus process, most statements received more than 75% agreement, leading to a refined protocol designed to enhance the safety and efficacy of HDMTX therapy. Supported by robust regulatory oversight, the UAE’s proactive mandate to stockpile key agents such as leucovorin, glucarpidase, bicarbonates, and diuretics ensures readiness for HDMTX toxicity emergencies. This highlights the importance of continued policy advocacy and institutional readiness to maintain stockpiling and healthcare infrastructure resilience. Additionally, there is a call for the integration of these consensus recommendations into national clinical guidelines, with established update cycles to keep protocols current.

Despite variability in global protocols and limited large-scale evidence, the consensus underscores the importance of early identification and timely intervention for DME, incorporating strategies such as hyperhydration, urine alkalization, leucovorin rescue, and glucarpidase use. This consensus provides a foundation for improving patient outcomes and guiding future practice in the UAE. Future research priorities should include validating recommendations through prospective studies, optimizing therapy with pharmacogenomic profiling, and investigating new therapeutic adjuncts to mitigate toxicity.
