# Supplementary material for: Consensus on managing delayed methotrexate elimination in high-dose therapy: insights from the Middle East
Source: Front Oncol. 2025 Nov 6;15:1660937. doi: 10.3389/fonc.2025.1660937 (PMC12631234; doi:10.3389/fonc.2025.1660937)
Supplement: Supplementary file 2 [file Table2.docx]

Supplementary Material

**1. Supplementary Table 1:** Initial consensus statements and their levels of agreement among the expert panels

| **S. No.** | **Consensus statements** | **Consensus reached** | **Level of evidence#** | **Grading#** | **Consensus attained** |
| --- | --- | --- | --- | --- | --- |
| **HDMTX regimen overview** | | | | | |
| 1 | IV administration of HDMTX (≥500 mg/m²) is pivotal in treating PCNSL, ALL, and specific subtypes of NHL due to its ability to penetrate the BBB at these doses (2,4). | A: 92.3%  N: 7.69% | 3a  1a | A  A | **HIGH** |
| 2 | MTX dosing and infusion times vary by diagnosis and patient characteristics, ranging from 0.5–5 g/m² over 4–36 hours in ALL to 8–12 g/m² over 4 hours in osteosarcoma (23). | A: 100% | 5 | C | **HIGH** |
| 3 | HDMTX remains the first-line treatment for ALL, NHL, and osteosarcoma and can be safely administered with supportive care measures (hyperhydration, urine alkalinization, high-dose leucovorin) to enhance MTX solubility and prevent toxicity (15,23). | A: 100% | 5  1a | C  A | **HIGH** |
| 4 | SCr level increase of ≥35.0 µM at 24 hours, 50% within 24 hours, or 25 µM/50% within 36 hours of HDMTX administration can be used as predictors for DME (3). | A: 92.3%  N: 7.69% | 5 | C | **HIGH** |
| 5 | HDMTX can be safely administered to patients with normal renal function through hyperhydration, urine alkalization, and pharmacokinetically guided leucovorin rescue (6). | A: 100% | 5 | C | **HIGH** |
| **Risk factors for DME and HDMTX toxicity** | | | | | |
| 6 | Patient-level risk factors for DME include BMI ≥25 kg/m², concomitant medications, urine pH <7.0, IV fluid intake <3 L/m²/24 h, third-space fluid collection, hepatic dysfunction, renal insufficiency, and diarrhea. Having three or more of these factors is linked to significantly poorer survival (25). | A: 92.3%  N: 7.69% | 4 | B | **HIGH** |
| 7 | Despite adherence to standard MTX protocols, most patients receiving HDMTX experienced DME, prolonged TTC , and increased LOS (8,42).  (**Was reframed**) | A: 46.15%  N: 15.38%  D: 38.36% | 3b  5 | B  C | **LOW** |
| 8 | HDMTX can cause severe toxicities, including nephrotoxicity, neurotoxicity, oral mucositis, neutropenia, and elevated liver enzymes; therefore, monitoring MTX levels and identifying risk factors are crucial (26). | A: 100% | 2b | B | **HIGH** |
| 9 | HDMTX administration results in AEs, with more than half of all patients experiencing mucositis and neutropenia (30). | A: 100% | 3b | C | **HIGH** |
| 10 | Higher rates of DME have been reported in patients with lymphoma receiving HDMTX compared to those with osteosarcoma (3,27). (**Removed**) | A: 53.84%  N: 38.46%  D: 7.69% | 5  3b | C  B | **LOW** |
| 11 | In pediatric patients with ALL or lymphoma, HDMTX therapy is associated with increased rates of DME (3,32).  (**Removed**) | A: 61.53%  N: 30.76%  D: 7.69% | 5  2b | C  B | **MODERATE** |
| 12 | HDMTX toxicity may be increased by coadministration of drugs that displace MTX from serum proteins or reduce its clearance, particularly TMP-SMX and NSAIDs (35). | A: 100% | 5 | C | **HIGH** |
| 13 | Presence of third space fluids, such as pleural effusions or ascites, contraindicate HDMTX administration due to the risk of prolonged MTX half-life and toxicity; drainage of these fluids before treatment is recommended (35). (**Was reframed)** | A: 76.92%  N: 15.38%  D: 7.69% | 5 | C | **HIGH** |
| 14 | A high BMI is significantly associated with an increased risk of AKI in patients receiving HDMTX; therefore, BMI should be considered in risk assessment and management strategies for these patients (4). | A: 84.61%  N: 15.38% | 3b | C | **HIGH** |
| 15 | In pediatric ALL patients, pre-infusion hypoalbuminemia (serum albumin <3.5 g/dL) is associated with increased HDMTX-related toxicities (37). (**Removed**) | A: 61.53%  N: 38.46% | 2b | C | **MODERATE** |
| 16 | Patient age and BSA are significant predictors of MTX clearance, with their effects primarily influencing the distribution and elimination phases of MTX kinetics (8). | A: 92.30%  N: 7.69% | 3b | B | **HIGH** |
| 17 | Serum MTX and SCr levels are key parameters for identifying potential HDMTX-induced AKI (23). | A: 100% | 5 | C | **HIGH** |
| 18 | Pretreatment KPS and renal function may significantly impact the outcomes of HDMTX therapy (38). | A: 84.61%  N: 7.69%  D: 7.69% | 5 | C | **HIGH** |
| 19 | Any grade of renal dysfunction, including mild impairment (e.g., Cr clearance <60 mL/min), may increase the risk of toxicity during HDMTX treatment (3). | A: 100% | 5 | C | **HIGH** |
| 20 | Furosemide has been identified as a risk factor for severe MTX-related renal toxicity and should be used cautiously when administering HDMTX (2,41).  (**Was reframed**) | A: 53.84%  N: 30.76%  D: 15.38% | 5  1b | C  B | **LOW** |
| 21 | Administering HDMTX in an outpatient setting improves bed utilization and reduces healthcare costs (2). (**Removed**) | A: 69.23%  N: 15.38%  D: 15.38% | 4 | C | **MODERATE** |
| **Supportive and preventive care** | | | | | |
| 22 | Supportive care must include measures to alkalinize urine and maintain adequate urinary flow to prevent MTX crystallization in the renal tubules (3). | A: 100% | 5 | C | **HIGH** |
| 23 | The hydration fluid should be supplemented with sodium bicarbonate to achieve a urine pH of ≥7; HDMTX should not be infused until this pH is reached (3). | A: 100% | 5 | C | **HIGH** |
| 24 | Hyperhydration (≥2.5 L/m² per 24 hours) with dextrose/saline and sodium bicarbonate should commence several hours prior to HDMTX administration and continue until MTX is cleared to nontoxic levels (3,43). | A:100% | 5  3b | C  B | **HIGH** |
| 25 | Increased hydration may mitigate the impact of age and BSA on MTX clearance by enhancing renal elimination; therefore, hyperhydration prior to the first cycle of HDMTX for older patients and those with higher BSA may help prevent DME (24). | A: 76.92%  N: 23.07% | 3b | B | **HIGH** |
| 26 | Loop diuretics or acetazolamide should be used to manage HDMTX toxicity to maintain diuresis and prevent fluid overload in patients with weight gain/fluid retention and in select cases of severe renal impairment (3). (**Was reframed**) | A: 61.53%  N: 30.76%  D: 7.69% | 5 | C | **MODERATE** |
| **Monitoring** | | | | | |
| 27 | In pediatric patients with ALL, an increased serum aspartate aminotransferase levels and age ≥10 years at diagnosis are independent risk factors for MTX neurotoxicity (46). (**Removed**) | A: 53.84%  N: 46.15% | 3b | C | **LOW** |
| 28 | UO within the first 24 hours had a significant impact on DME, TTC, and LOS in patients receiving HDMTX (8). | A: 92:30%  N: 7.69% | 3b | C | **HIGH** |
| 29 | An increase in SCr level within 24–36 hours after starting HDMTX may serve as an early indicator of DME (3). | A: 100% | 5 | C | **HIGH** |
| 30 | During HDMTX administration, maintaining UO at >100 mL/m²/h and urine pH ≥7, while avoiding weight gain is important (3,11). | A: 100% | 5  5 | C  C | **HIGH** |
| 31 | Cr and/or GFR should be monitored every 24 hours starting 24 hours after HDMTX administration, with closer monitoring, including cystatin C, if DME is suspected (3,11). | A: 84.61%  N: 7.69%  D: 7.69% | 5  5 | C  C | **HIGH** |
| 32 | Patient discharge can be considered when serum MTX is <0.1 µmol/L with stable renal function and electrolytes and no significant fluid overload or on Day 3 after HDMTX infusion if MTX kinetics at 48 hours are favorable (serum MTX <1 µmol/L) and SCr levels are stable (3). | A: 100% | 5 | C | **HIGH** |
| 33 | Developing clinical decision support tools, such as MTXPK.org, can optimize model-informed prediction and timely intervention for DME before starting HDMTX administration by utilizing individualized patient data, including demographics, SCr levels, and real-time drug concentrations (49) | A: 76.92%  N: 23.07% | 2b | D | **HIGH** |
| 34 | At 36 hours after MTX administration, leucovorin rescue should be initiated with 15 mg/m² for low-severity adults and 30 mg/m² for moderate- to high-severity adults, and leucovorin should be discontinued when plasma MTX concentration drops below 0.1 µmol/L (50). **(Was reframed)** | A: 84.61%  N: 15.38% | 3b | C | **HIGH** |
| 35 | Most clinics use immunoassays to measure serum MTX levels; however, these methods do not reliably distinguish between MTX and its metabolites and glucarpidase treatment may interfere with the results of these assays (3). **(Was reframed)** | A: 76.92%  N: 23.07% | 5 | C | **HIGH** |
| 36 | MTX levels are monitored using immunoassays, which are often conducted on automated analyzers; however, they incur high initial and recurring costs along with refrigeration requirements, making them complex and expensive (51). | A: 84.61%  N: 15.38% | 3a | B | **HIGH** |
| 37 | Chromatographic techniques are considered the gold standard for monitoring MTX levels, but they require significant initial investment, specially trained personnel, and have longer turnaround times for results (51). | A: 92.38%  N: 7.69% | 3a | C | **HIGH** |
| **Emergent care of DME and HDMTX toxicity** | | | | | |
| 38 | Upon detection of HDMTX-induced AKI, initial supportive measures include urine alkalinization, fluid hydration, and high-dose leucovorin (23). | A: 100% | 5 | C | **HIGH** |
| 39 | Despite initial supportive care, if HDMTX toxicity persists, emergent care is necessary, which includes increased urine alkalinization, enhanced fluid hydration, and higher doses of leucovorin. Additionally, dialysis methods and glucarpidase may be considered (23). | A: 100% | 5 | C | **HIGH** |
| 40 | Patients with MTX toxicity receiving HFHD experience prolonged hospitalization, increased ICU use, high mortality, and significant MTX rebound after treatment, necessitating additional clearance sessions (42,52). | A: 92.30%  N: 7.69% | 1a  1a | A  A | **HIGH** |
| 41 | Intensive HFHD can effectively clear MTX in patients with ESRD (54). | A: 84.61%  N: 15.38% | 4 | C | **HIGH** |
| 42 | Various leucovorin rescue protocols for DME are available, typically initiated 24–36 hours post-MTX administration, with leucovorin administered every 6 hours at doses adjusted based on serum MTX levels (3). | A: 100% | 5 | C | **HIGH** |
| **Glucarpidase use** | | | | | |
| 43 | Glucarpidase is typically administered in cases of DME and HDMTX toxicity with renal deterioration, such as a >50% increase in SCr levels within 24–48 hours (23). | A:100% | 5 | C | **HIGH** |
| 44 | Since glucarpidase mitigates the risk of acute renal failure by correcting DME in adult and pediatric cancer patients, its use allows for the continuation of HDMTX therapy without additional toxicity (57). | A: 92.30%  N: 7.69% | 3b | B | **HIGH** |
| 45 | Leucovorin, a substrate for glucarpidase, should be discontinued at least 2 hours before and resumed no sooner than 2 hours after glucarpidase infusion, continuing until serum MTX levels are undetectable (3,11). | A: 92.30%  D: 7.69% | 5  5 | C  C | **HIGH** |
| 46 | A dose of glucarpidase at 50 U/kg is effective and well-tolerated in pediatric, adolescent, and young adult patients with DME without renal dysfunction (58). | A: 100% | 3a | C | **HIGH** |
| 47 | MTX levels should be monitored after glucarpidase administration, ideally using an HPLC-based assay, until undetectable (3). | A: 84.60%  N: 15.38% | 5 | C | **HIGH** |
| 48 | In HDMTX-AKI patients, glucarpidase treatment within 60 hours significantly increases the odds of renal recovery, recovery from neutropenia, and normalization of liver enzymes, particularly enhancing renal recovery (61). | A: 92.30%  N: 7.69% | 2b | C | **HIGH** |
| 49 | Glucarpidase may be considered in cases of impaired renal function when plasma MTX concentrations are 2 standard deviations above the mean (as per MTXPK.org) or if plasma MTX levels exceed 50 μmol/L at 24 hours, 30 μmol/L at 36 hours, 10 μmol/L at 42 hours, or 5 μmol/L at 48 hours (3). | A: 92.30%  N: 7.69% | 5 | C | **HIGH** |
| 50 | Adult cancer patients treated with glucarpidase have lower inpatient and 90-day mortality rates than those who do not receive glucarpidase (including those on hemodialysis). They also have shorter overall hospital LOS and ICU stays (42,60). | A: 84.60%  N: 15.38% | 3b  2c | C  C | **HIGH** |
| 51 | Administering glucarpidase within 72 hours of MTX significantly reduces the risk of severe MTX toxicity, with the recommended window being 48 to 60 hours after the start of the HDMTX infusion (62,63). | A: 100% | 5c  1a | C  C | **HIGH** |
| 52 | Timely glucarpidase use is more cost-effective than delayed administration or hemodialysis (60). | A: 84.60%  N: 15.38% | 2b | D | **HIGH** |
| 53 | Despite its effectiveness in lowering blood MTX levels, glucarpidase has minimal impact on intracellular MTX levels. Therefore, high-dose folinic acid must also be administered to manage intracellular MTX (6,28). | A: 76.92%  N: 23.07% | 5  5 | C  C | **HIGH** |
| 54 | Within 15–20 minutes of glucarpidase administration, MTX levels typically drop to 20% (as tested by immunoassay) and 1% (as tested by HPLC) of the original levels (11,59). | A: 76.92%  N: 23.07% | 4  5 | B  C | **HIGH** |

**A:** Agree; **N:** Neutral and **D:** Disagree

**ALL**: Acute lymphoblastic leukemia; **AE:** Adverse event; **AKI:** Acute kidney injury; **BBB:** Blood–brain barrier; **BMI:** Body mass index; **BSA:** Body surface area; **Cr:** Creatinine; **DME:** Delayed methotrexate elimination; **ESRD**: End-stage renal disease; **GFR:** Glomerular filtration rate; **HFHD:** High-flux hemodialysis; **HDMTX:** High-dose methotrexate; **HPLC:** High-performance liquid chromatography; **ICU:** Intensive care unit; **IV:** Intravenous; **KPS:** Karnofsky performance status; **LOS**: Length of stay; **MTX:** Methotrexate; **NHL:** Non-Hodgkin lymphoma; **NSAID:** Nonsteroidal anti-inflammatory drug; **PCNSL:** Primary central nervous system lymphoma; **SCr:** Serum creatinine; **TTC:** Time to clearance; **TMP-SMX:** Trimethoprim/sulfamethoxazole; **UO:** Urine output.

**#**Level of evidence: **Oxford Centre for Evidence-Based Medicine: Levels of Evidence (March 2009)**
