# Supplementary material for: Consensus on managing delayed methotrexate elimination in high-dose therapy: insights from the Middle East
Source: Front Oncol. 2025 Nov 6;15:1660937. doi: 10.3389/fonc.2025.1660937 (PMC12631234; doi:10.3389/fonc.2025.1660937)
Supplement: Supplementary file 3 [file Table3.docx]

**Supplementary Table 3: Assay methods for measuring serum MTX levels**

| Assay Type | Description / Features | Advantages | Limitations | References |
| --- | --- | --- | --- | --- |
| Immunoassay | Measures serum MTX concentrations using antibody-based detection. | High automation; rapid results; widely used in clinical laboratories | Can cross-react with MTX metabolites (DAMPA, glutamate); expensive; requires refrigeration | (3,51) |
| Chromatography (HPLC/LC-MS) | Separates MTX from metabolites using high-performance or liquid chromatography techniques. | Gold standard; high specificity and accuracy; minimal interference from metabolites | Requires specialized equipment and trained personnel; longer turnaround time; high initial cost | (51) |
| Practical Notes | Immunoassays are commonly used in routine laboratories; chromatographic methods are reserved for precise measurement or research applications. | | | |

**MTX –** methotrexate; **DAMPA** – 2,4-diamino-N^10^-methylpteroic acid; **HPLC** – High-performance liquid chromatography; **LC-MS** – Liquid chromatography mass spectrometry.
